# Validation and Cross-Cultural Adaptation of the Diabetes Self-Management Questionnaire (DSMQ) and the Social Phobia Inventory (SPIN) in Romanian Patients with Diabetes Mellitus

**DOI:** 10.3390/medicina58121823

**Published:** 2022-12-11

**Authors:** Laura Diaconu, Laura Gaita, Bogdan Timar, Loredana Deaconu, Sandra Lazar, Romulus Timar, Simona Popescu

**Affiliations:** 1 Second Department of Internal Medicine, “Victor Babes” University of Medicine and Pharmacy, 300041 Timisoara, Romania; 2 “Pius Brinzeu” Emergency Hospital, 300723 Timisoara, Romania; 3 Centre for Molecular Research in Nephrology and Vascular Disease, “Victor Babes” University of Medicine and Pharmacy, 300041 Timisoara, Romania

**Keywords:** diabetes self-management, social phobia, mental health, diabetes care, instrument validation

## Abstract

*Background and Objectives*: Anxiety disorders are common in individuals with diabetes mellitus (DM) and have a negative impact on diabetes-related self-management and, therefore, on patients’ evolution and prognosis. In this context, it becomes necessary to accurately and easily assess anxiety and self-management behaviours. Thus, the aim of this research was translation and cultural adaptation for Romanian patients and validation of two instruments used for assessing diabetes self-management and anxiety, namely the Diabetes Self-Management Questionnaire (DSMQ) and the Social Phobia Inventory (SPIN). *Materials and Methods*: The Summary of Diabetes Self-Care Activities Questionnaire (SDSCA) and the DSMQ instruments for assessing diabetes self-management, as well as the Generalised Anxiety Disorder Scale (GAD-7) and the SPIN instruments for assessing anxiety, have been administered to 117 patients from Timisoara, Romania, previously diagnosed with DM. *Results*: The SPIN has proven to have good internal consistency, excellent acceptability of its questions without non-responders and a median completion time of 3 min and 10 s, an excellent test–retest performance (Spearman’s rho = 0.971, *p* < 0.001 between two administrations of the test) and good validity in comparison with the GAD-7, a previously validated and comprehensive instrument. The DSMQ has also proven to have acceptable internal consistency, excellent acceptability of its questions without non-responders and a median completion time of 2 min and 28 s; however, it has shown a weak, positive correlation without statistical significance in comparison with the SDSCA, a previously validated questionnaire. *Conclusions*: The SPIN, translated in Romanian and culturally adapted, is a valid tool for the screening of social phobias in individuals with DM. The DSMQ requires additional data for its validation in DM patients from Romania.

## 1. Introduction

The prevalence and impact of noncommunicable diseases have increased dramatically in the past decades. Among these, diabetes mellitus (DM) has become a challenge for healthcare systems all around the globe with approximately 537 million persons worldwide living with diabetes and with an estimated increase to 783 million individuals by 2045 [1]. It is also known that the 21st century has witnessed a rise in the prevalence of psychiatric diseases, with significant negative effects on morbidity and on patients’ quality of life, with some of these being frequent comorbidities in patients with DM [2].

Among mental health conditions, anxiety disorders are common in individuals with DM in such a degree that some authors consider this metabolic disease as a risk factor for anxiety symptoms, while the most frequently encountered types of anxiety in patients with DM are generalised anxiety disorder and social phobia [3,4,5,6,7].

Several trials have proven the negative impact of psychiatric diseases on diabetes-related self-care and, subsequently, on glycaemic control and even on the risk of mortality of the affected patients [8,9,10,11,12]. This association is of paramount importance since it is already well known that diabetes self-management education and support represents one of the most important aspects of diabetes treatment strategy and that the improvement of self-care behaviours can lead to a reduced risk of chronic degenerative complications, such as microangiopathy, macroangiopathy and neuropathy, acute metabolic complications and to a more favourable evolution of the disease [13,14,15,16,17,18,19,20].

Therefore, it becomes evident that for successful, comprehensive and interdisciplinary management of patients with DM it is essential to accurately assess the diabetes-related self-care activities early in all patients with DM and also to determine the presence and severity of additional psychological conditions, including anxiety-related diseases. Several accessible, easy-to-use tools are available for evaluating both diabetes self-management and anxiety disorders; however, it is known that different cultures can have different metabolic, mental and behavioural characteristics and, thus, it is necessary to validate these instruments before drawing conclusions.

With these premises, our study aimed to translate, culturally adapt for Romanian patients and validate an instrument used for assessing diabetes self-management, the Diabetes Self-Management Questionnaire (DSMQ), respectively an instrument used for assessing an anxiety disorder - namely the Social Phobia Inventory (SPIN).

## 2. Materials and Methods

### 2.1. Study Design and Patients

In this cross-sectional, non-interventional study, we included 117 patients, diagnosed previously with type 1 and type 2 DM, hospitalised in the Diabetes Clinic of the “Pius Brinzeu” Emergency County Hospital, Timisoara, Romania. The subjects were enrolled according to a population-based, consecutive-case principle. All patients provided written informed consent before performing any study procedure or activity. Since this is a cross-sectional, non-interventional study, according to the local protocols it had to be approved only by the local Ethics Committee. The exclusion criteria have been the inability to provide informed consent and to provide accurate anamnestic data or any diseases or parameters which, in the investigators’ opinions, could lead to biases in the study results.

### 2.2. Clinical, Anthropometric and Laboratory Data

Detailed characteristics of the studied sample, presented in Table 1, have been collected from the patients’ medical records. HbA1c, uric acid, lipid profile, urinary albumin/creatinine ratio, glomerular filtration rate, TSH and FT_4_ measurements were performed after at least 12 h of fasting, using standardised methods. The diagnosis of retinopathy was established using a fundoscopic examination, while the diagnosis of diabetic neuropathy was established using standardised sensation tests. The patients’ characteristics at enrolment were very similar to the ones of the general population of Romanian patients with DM and, thus, the studied sample was highly representative of this population.

### 2.3. Diabetes Self-Management

The quality of diabetes-related self-management activities was assessed using two instruments—the Summary of Diabetes Self-Care Activities Questionnaire (SDSCA) and the Diabetes Self-Management Questionnaire (DSMQ).

The SDSCA is a validated self-report tool for measuring diabetes self-management in adults. Eleven items assess the frequencies of multiple diabetes-related and lifestyle activities during the previous week or month. Patients are asked to mark the number of days per week in which they had a healthy or unhealthy diet (4 questions), in which they participated in specific types of physical activity (2 questions), in which they measured their blood glucose levels (2 questions), in which they performed foot-care activities (2 questions) and in which they smoked (1 question + if yes, the number of cigarettes per day). After receiving written permission from the Oregon Research Institute to use the SDSCA, the included patients were instructed to answer the 11 items of this instrument [21].

The other questionnaire, the DSMQ, an instrument still requiring validation in the population of Romanian patients with DM, is also used to assess behaviours associated with self-care for this noncommunicable disease. It consists of 16 items that cover different aspects of self-care in these individuals: glucose-level management, dietary control, physical activity and patient–physician interaction. Subjects are asked to rate the extent to which each item applied to them in the past 8 weeks (0—does not apply to me, 3—applies to me very much). After receiving written permission from the Mapi Research Trust, the included patients were asked to answer the items twice (the second time with a day in between the responses) [22].

### 2.4. Anxiety Assessment

Anxiety was evaluated using two instruments—the Generalised Anxiety Disorder Scale (GAD-7) and the Social Phobia Inventory (SPIN).

The GAD-7 is an instrument useful in assessing anxiety. It consists of 7 items and subjects are asked to mark the degree in which the described feelings bothered them in the past 2 weeks (0—not at all, 3—nearly every day). This validated tool was already available in Romania and patients were instructed to answer every item [23].

The SPIN, still requiring validation in the population of Romanian patients with DM, is used in screening for and the measuring of the severity of social anxiety disorder. It includes 17 items and a higher score indicates more severe symptoms such as fear, avoidance and others experienced by subjects in various social situations. The answers are arranged on a five-point scale describing the extent in which each statement applied to the patient in the past week (0—not at all, 4—extremely). The questionnaire was translated in Romanian after receiving written permission from the authors and then patients were asked to answer the items twice (the second time with a day in between the responses) [24].

### 2.5. Statistical Analysis

Data have been recorded and analysed using SPSS v.17 (SPSS Inc, Chicago, IL, USA). The results are presented as mean ± standard deviation for numerical variables with Gaussian distribution and median [interquartile range] for numerical variables with non-parametric distribution, respectively, as a percentage from the subgroup’s total. To assess the normality of the numerical variables’ distribution, the Shapiro–Wilk method was used.

The statistical significance of differences between groups was assessed using the unpaired t-Student test (numerical variables with Gaussian distribution) and the Mann–Whitney U-test (numerical variables with non-parametric distribution), respectively, and the Chi-Square test for nominal variables.

In validating the aforementioned questionnaires, we used three analyses: the reliability (internal consistency), the reproducibility and the external validity.

The sample size of the studied lot was calculated prior to enrolment, based on previous similar literature data, to provide a confidence level of 95% and a statistical power of at least 80%. In this study, a *p*-value lower than 0.05 was considered to be the threshold of statistical significance.

## 3. Results

### 3.1. Diabetes Self-Management Questionnaire (DSMQ)

#### 3.1.1. DSMQ Score Distribution

**Figure 1:**
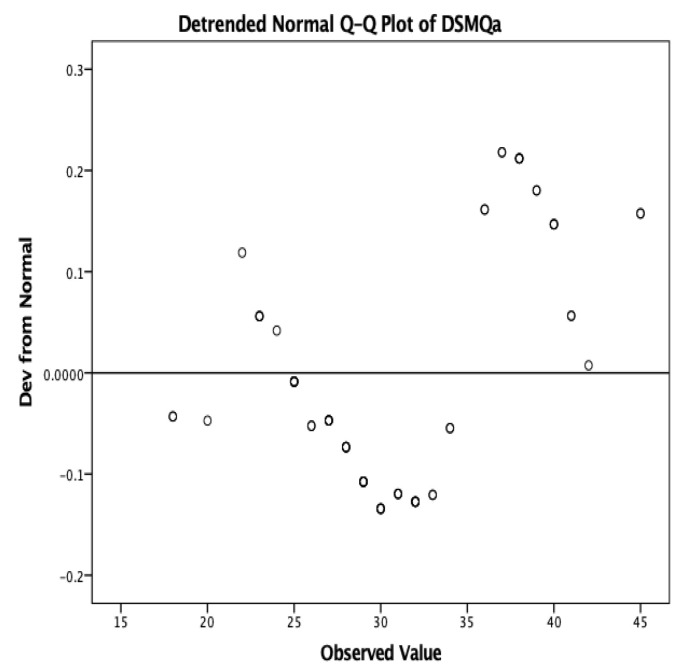
Distribution of the scores obtained in the DSMQ (DSMQa—initial evaluation).

In both initial and follow-up administrations of the DSMQ, 117 responses were recorded. The median scores were 31.13 for the initial evaluation and 22.77 for the final evaluation. The distribution of the scores was unimodal, Gaussian, Shapiro–Wilk = 0.977, *p* = 0.297 (Figure 1).

#### 3.1.2. Acceptability, Ceiling and Floor Effect

At both initial and follow-up evaluation, all the subjects (n = 117) responded to all of the DSMQ items. The median time needed for completion was 2 min and 28 s (minimum 1 min and 45 s, maximum 3 min and 10 s). Regarding the total score, there was no aggregation at the top end of the scale, while the maximum number of points has not been reached by any of the participants. The minimum possible score was also in the desired range, without any participants scoring the minimum number of points; thus, the floor and ceiling effects have been ruled out.

#### 3.1.3. Reliability

The internal consistency of the DSMQ for the Romanian population of patients with DM was assessed using Cronbach’s alpha method. The questionnaire has proven to have acceptable internal consistency with Cronbach’s α = 0.683 [0.555–0.787] 95% CI in the first evaluation and Cronbach’s α = 0.794 [0.720–0.866] 95% CI in the second evaluation. At the same time, for both instances there was a high inter-item correlation with a low inter-item covariance (Table 2).

#### 3.1.4. External Validity

**Figure 2:**
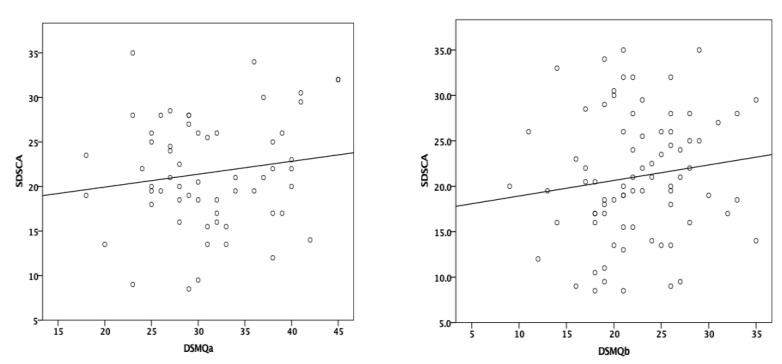
Correlation between the DSMQ and SDSCA (DSMQa—initial evaluation; DSMQb—follow-up evaluation).

The DSMQ has shown a weak, positive correlation with a previously validated questionnaire, the SDSCA, in both first (Spearman’s rho = 0.070) and second (Spearman’s rho = 0.162) measurements, however without statistical significance (*p* = 0.595, *p* = 0.156, respectively) (Figure 2).

#### 3.1.5. Reproducibility

**Figure 3:**
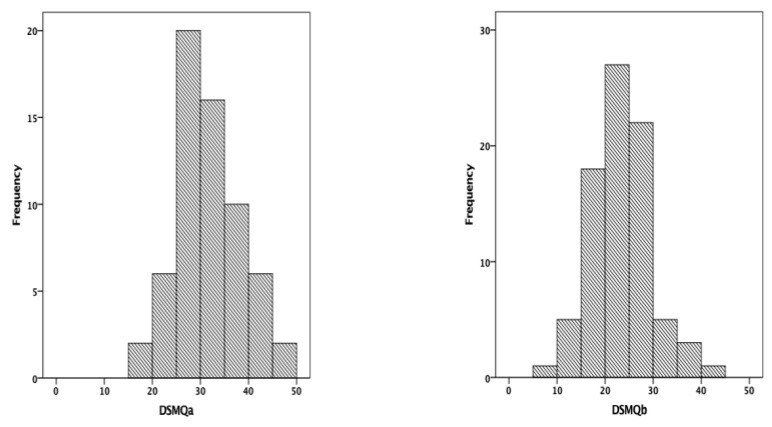
The test–retest assessment of the DSMQ (DSMQa—initial evaluation; DSMQb—follow-up evaluation).

With the test–retest assessment, a statistically significant, moderate, negative correlation was shown between the two measurements of the DSMQ (Spearman’s rho = −0.357, *p* = 0.004), while the medians of the scores that have resulted from the two measurements have been 30 and 22, respectively (Figure 3).

### 3.2. Social Phobia Inventory (SPIN)

#### 3.2.1. SPIN Score Distribution

**Figure 4:**
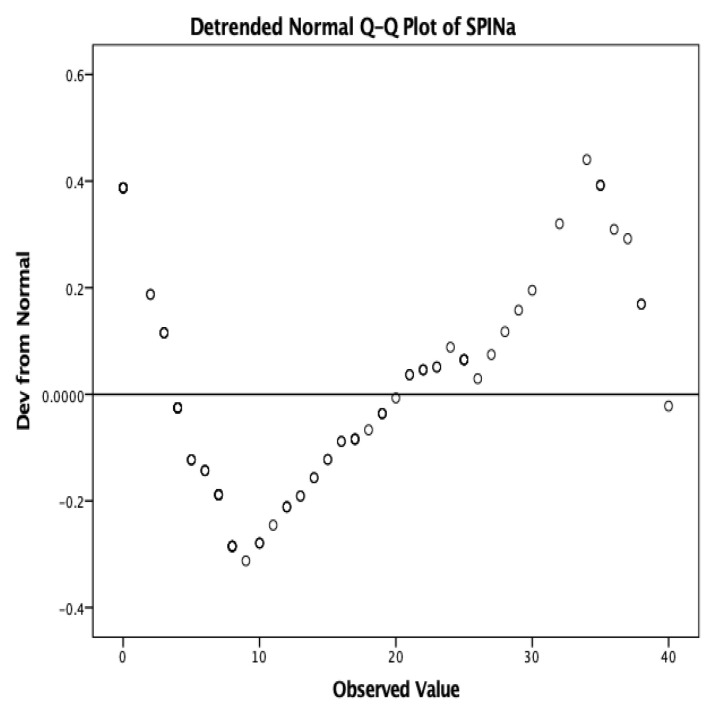
Distribution of the scores obtained in the SPIN (SPINa—initial evaluation).

In both the first and second administrations of the SPIN, 117 responses were received. The median scores were 15.10 for the initial evaluation and 15.73 for the final evaluation. The distribution of the scores was unimodal, non-Gaussian, Shapiro–Wilk = 0.936, *p* < 0.001 (Figure 4).

#### 3.2.2. Acceptability, Ceiling and Floor Effect

In both the first and second evaluation, every subject (n = 117) responded to all of the SPIN items. The median time for completing the questionnaire was 3 min and 19 s (minimum 2 min and 10 s, maximum 3 min and 48 s). Regarding the total score, no aggregation at the top end of the scale was recorded, while the maximum number of points has not been reached by any of the participants. Furthermore, there were not any participants that scored the minimum number of points; thus, the floor and ceiling effects have been excluded.

#### 3.2.3. Reliability

The internal consistency of the SPIN for Romanian patients with DM was assessed using Cronbach’s alpha method. The questionnaire has proven to have good internal consistency with Cronbach’s α = 0.892 [−0.044–1.098] 95% CI in the first measurement and Cronbach’s α = 0.898 [−0.009–1.162] 95% CI in the second measurement. At the same time, for both instances there was a high inter-item correlation and a low inter-item covariance (Table 3).

#### 3.2.4. External Validity

**Figure 5:**
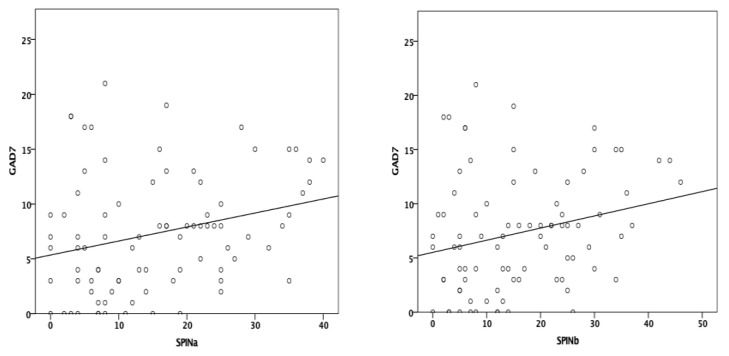
Correlation between the SPIN and GAD-7 (SPINa—initial evaluation; SPINb—follow-up evaluation).

The SPIN has shown a statistically significant, weak, positive correlation with a previously validated questionnaire, the GAD-7, in both the initial (Spearman’s rho = 0.274, *p* < 0.001) and follow-up (Spearman’s rho = 0.256, *p* = 0.018) measurements (Figure 5).

#### 3.2.5. Reproducibility

**Figure 6:**
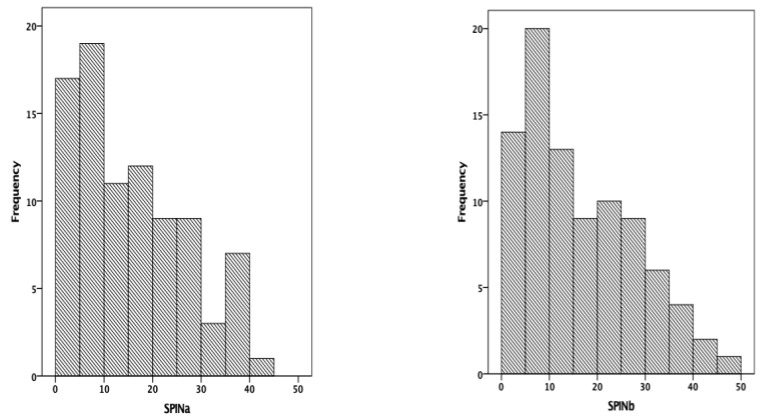
The test–retest assessment of the SPIN (SPINa—initial evaluation; SPINb—follow-up evaluation).

With the test–retest assessment, a strong positive correlation with statistical significance was shown between the two administrations of the SPIN (Spearman’s rho = 0.971, *p* < 0.001), while the median of the scores that has resulted from the two measurements has been 13 and the differences between the two measurements did not have statistical significance (Figure 6).

## 4. Discussion

The results of this research have shown that the SPIN could become a useful instrument in the diagnosis and assessment of the severity of social phobia in patients with DM in Romania since it has proven to have good internal consistency, excellent acceptability and a test–retest reliability that is similar to other more complex instruments. Moreover, all of the enrolled patients have answered all of its items, in both measurements, in a short duration of time, with a median of 3 min and 19 s.

The validity of the SPIN was also proven to be good since a positive correlation with statistical significance was shown between the scores resulting from this questionnaire and from the GAD-7, a previously validated instrument. The weak correlation between these results could be explained by the two different conditions assessed by the two self-administered questionnaires, namely social phobia and generalised anxiety disorder. However, the aforementioned results and the validation of the SPIN in multiple other trials in different countries on various continents support the usage of this instrument for the screening of social phobia in patients—including Romanian patients—with DM [25,26].

Similar results of this study have been shown with regard to the DSMQ, an instrument designed to assess diabetes self-management. However, although this questionnaire has proven to have acceptable internal consistency and has been used with ease by all of the enrolled patients, its weak positive correlation with a previously validated tool, namely the SDSCA, has not reached statistical significance, hence the necessity of further evidence before stating its validity for Romanian patients with DM. This finding could be explained by the difficulty in assessing diabetes self-management with a standardised test and not through a partially guided interview, direct observation or interpretation of glycaemic control or cardiometabolic risk factors. Nevertheless, the DSMQ has already been validated in diverse subpopulations across the globe, with more favourable results in various studies showing data that encourage future research regarding this valuable tool [27,28].

The strengths of this research include the consecutive enrolment of the subjects that resulted in a heterogenous cohort of individuals, with or without social phobia, with diverse values of age, metabolic parameters, DM evolution and complications, antihyperglycemic agents and diabetes self-care behaviours. Moreover, this study is the first of its kind in Romania, and the first one that validated the translated and culturally adapted SPIN version, while being the first step in validating the DSMQ. Furthermore, we have addressed sources of potential bias through using standardised methods of assessing complications of diabetes and characteristics of patients, through a clearly defined study population with enrolment according to a consecutive-case principle, with clear inclusion and exclusion criteria, through using well-known instruments in evaluating anxiety and diabetes self-management and through not interfering with the completion of the four questionnaires by the patients.

The main drawback of this research consists of the potential discrepancy between the general attributes of the enrolled patients and the population of individuals with DM from Romania, since we only included patients admitted in the Diabetes Clinic of the “Pius Brinzeu” Emergency County Hospital, Timisoara, Romania. However, the sample size was calculated to provide a statistical power level that would allow the inference of the results for the entire previously mentioned population.

The importance of this study lies in the increasing options regarding validated questionnaires that can be used to assess anxiety disorders and diabetes self-management accurately and early in the management of patients with DM from Romania, in a non-time-consuming and even self-administered manner, an essential characteristic when including new procedures into routine clinical practice. The analysis of self-management in patients with DM could have beneficial effects in the prevention of acute and chronic complications and in slowing the progression of chronic complications that are already present, while the detection of social phobia and other anxiety-related conditions could contribute to an improved prognosis and quality of life of DM individuals through an interdisciplinary approach.

## 5. Conclusions

The SPIN self-administered questionnaire, translated in Romanian and culturally adapted, is a valid instrument for the screening of social phobia in individuals with DM, while the DSMQ, that can be used for evaluating diabetes self-management behaviours, requires additional data for its validation. The SPIN has proven to have excellent acceptability and reliability and a test–retest performance comparable to other more complex tools that can be used to assess anxiety disorders and, thus, is a valuable and reliable questionnaire that can be used in daily clinical practice.

## Figures and Tables

**Table 1:** Patients’ general characteristics at enrolment.

Parameter	Value
Male gender ^a^	51 (43.5%)
Age ^b^ (years)	60 [±13.5]
BMI ^b^ (kg/m^2^)	31 [8]
eGFR ^c^ (mL/min/1.73 m^2^)	73.87 ± 25.36
UACR ^b^ (mg/g)	20.5 [7.28]
TC ^c^ (mg/dL)	181.24 ± 61.86
LDLc ^c^ (mg/dL)	103.43 ± 48.06
HDLc ^b^ (mg/dL)	42 [15.5]
TG ^b^ (mg/dL)	131 [94.8]
HbA1c ^c^ (%)	8.5 ± 1.64
UA ^b^ (mg/dL)	5.4 [2.9]
TSH ^b^ (mU/L)	1.96 [1.49]
FT4 ^b^ (ng/dL)	14.9 [3.04]

^a^ Categorical variables. Results are presented as number of cases and (percentage from the total). ^b^ Numerical variables with non-parametric distribution. Results are presented as median and [interquartile range]. ^c^ Numerical variables with Gaussian distribution. Results are presented as mean ± standard deviation. BMI—body mass index; eGFR—estimated glomerular filtration rate; UACR—urinary albumin/creatinine ratio; TC—total cholesterol; LDLc—low density lipoprotein cholesterol; HDLc—high density lipoprotein cholesterol; TG—triglycerides; HbA1c—haemoglobin A1c; UA—uric acid; TSH—thyroid stimulating hormone; FT4—free thyroxine.

**Table 2:** Inter-item covariances and correlations in the DSMQ.

	First Measurement	Second Measurement
	(n = 117)	(n = 117)
Inter-item covariances	0.112	0.198
Inter-item correlations	0.114	0.198

**Table 3:** Inter-item covariances and correlations in the SPIN.

	First Measurement	Second Measurement
	(n = 117)	(n = 117)
Inter-item covariances	0.364	0.418
Inter-item correlations	0.329	0.348

## Data Availability

Data are available on request due to privacy restrictions. The data presented in this study are available on request from the corresponding author after the request’s approval by the hospital’s ethics committee. The data are not publicly available due to privacy restrictions according to the hospital’s internal regulations.
